# Quantitative summaries of treatment effect estimates obtained with network meta-analysis of survival curves to inform decision-making

**DOI:** 10.1186/1471-2288-13-147

**Published:** 2013-12-01

**Authors:** Shannon Cope, Jeroen P Jansen

**Affiliations:** 1 Mapi Group, 33 Bloor Street East, Suite 1300, Toronto M4W 3H1, Canada; 2 Tufts University School of Medicine, Boston MA, USA

**Keywords:** Network meta-analysis, Rank, Probabilities, Survival

## Abstract

**Background:**

Increasingly, network meta-analysis (NMA) of published survival data are based on parametric survival curves as opposed to reported hazard ratios to avoid relying on the proportional hazards assumption. If a Bayesian framework is used for the NMA, rank probabilities associated with the alternative treatments can be obtained, which directly support decision-making. In the context of survival analysis multiple treatment effect measures are available to inform the rank probabilities.

**Methods:**

A fractional polynomial NMA of overall survival in advanced melanoma was performed as an illustrative example. Rank probabilities were calculated and presented for the following effect measures: 1) median survival; 2) expected survival; 3) mean survival at the follow-up time point of the trial with the shortest follow-up; 4) hazard or hazard ratio over time; 5) cumulative hazard or survival proportions over time; and 6) mean survival at subsequent time points. The advantages and disadvantages of the alternative measures were discussed.

**Results:**

Since hazard and survival estimates may vary over time for the compared interventions, calculations of rank probabilities for an NMA of survival curves may depend on the effect measure. With methods 1–3 rank probabilities do not vary over time, which are easier to understand and communicate than rank probabilities that vary over time as obtained with methods 4–6. However, rank probabilities based on methods 4–6 provide useful information regarding the relative treatment effects over time.

**Conclusions:**

Different approaches to summarize results of a NMA of survival curves with rank probabilities have pros and cons. Rank probabilities of treatment effects over time provide a more transparent and informative approach to help guide decision-making than single rank probabilities based on collapsed measures, such as median survival or expected survival. Rank probabilities based on survival proportions are the most intuitive and straightforward to communicate, but alternatives based on the hazard function or mean survival over time may also be useful.

## Background

Randomized controlled trials (RCTs) are often used to inform healthcare decisions [1-4]. In the absence of a head-to-head or direct comparison, indirect treatment comparisons provide a useful alternative [2,5-9]. An evidence base that consist of multiple RCTs where each trial has at least one intervention in common with another can be synthesized by means of a network meta-analysis (NMA). This method provides pooled estimates of available direct comparisons, indirect comparisons of pairwise contrasts for which no head-to-head RCT is available, and a synthesis of consistent direct and indirect evidence, resulting in more precise treatment effect estimates [3,10]. NMAs provide a comprehensive synthesis of the evidence from RCTs for multiple treatments useful for decision-makers to assess whether a new treatment should be adopted or whether additional evidence is required in the presence of uncertainty [11].

Meta-analyses or NMAs can be performed in a frequentist or a Bayesian framework. The result of a frequentist meta-analysis comparing treatments A and B is an estimate of the treatment effect (i.e. difference between A and B) as well as an associated p-value. The p-value indicates whether the results are statistically ‘significant’ or ‘non-significant’. If results are significant, the probability of erroneously rejecting the null hypothesis is judged to be small enough given the observed data. For example, if treatment A is considered significantly better than treatment B then the difference between the treatments is considered extreme enough to suggest that there is only a small probability (<5%) of incorrectly rejecting the null hypothesis. Therefore the p value reflects the probability of observing such a treatment difference assuming the null hypothesis is true. However, decision-makers are interested in minimizing the risk of an unsupported positive interpretation as well as the risk of overlooking a true difference. In other words, probabilities associated with the alternative hypothesis (i.e. A is better than B) are of interest but cannot be deduced from a frequentist analysis. Moreover, for an analysis of more than two treatments, p values resulting from a frequentist analysis associated with each pairwise comparison do not provide a straightforward interpretation of the relative efficacy or safety of the alternative interventions for decision-makers.

By using a Bayesian NMA it is possible to calculate the probability of being the best treatment out of all those treatments assessed with respect to the outcome of interest. This approach combines a prior probability distribution (representing a prior belief of the possible values for parameter) with a likelihood distribution of the observed effect, resulting in a posterior probability distribution [12]. With Monte Carlo simulations the probability that a treatment is best is calculated based on the proportion of cycles during the sampling process where a treatment ranks first of out all the treatments included in terms of the treatment effect size [13]. Similarly, it is possible to calculate the probability of being the second best treatment, third best treatment, etc., up until the probability of being worst treatment out of those assessed. These probability statements offer an intuitive summary of the joint posterior distribution of the effect sizes for all the included treatments, which naturally facilitates decision-making [13].

Salanti et al. have proposed several methods to present rank probabilities of treatments. Given the challenge of efficiently summarizing results from an analysis involving multiple pairwise comparisons, probabilities are positioned as a useful alternative to ‘p values’ resulting from a frequentist analysis. The importance of presenting a complete overview of the probabilities associated with each ranking is emphasized to avoid the over-interpretation of the probabilities associated with being the ‘best’ treatment, which necessitates a more comprehensive approach to present the information. Therefore, several different approaches are proposed by Salanti et al. to summarize the probabilities in a clear a concise manner. However, all of the methods implicitly assume that the treatment effects are constant over time [13].

In many RCTs the endpoint of interest is the time to the occurrence of a certain event, such as time to progression, progression-free survival, or overall survival. The synthesis of published results across different studies by means of an NMA is typically based on the constant hazard ratio (HR). However, it has been recognized that an NMA that relies on the proportional hazards assumption is biased if the survival curves or hazard functions of competing interventions cross [14-17]. Recently NMA models for survival data have been presented that do not assume a constant HR but allow the relative treatment effects to vary over time [14-16]. Such analyses can result in time-varying HRs, survival proportions over time, and expected survival by treatment. In order to apply the methods proposed by Salanti et al. to these analyses it is important to acknowledge that treatment effects may vary over time.

In this paper we discuss alternative approaches to present rank probabilities in the context of a Bayesian NMA of parametric survival curves.

## Methods

### Motivating example

#### Evidence base

**Figure 1:**
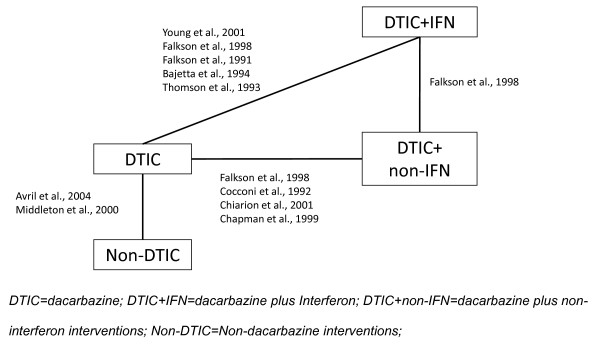
Network of randomized controlled trials.

As an illustrative example the efficacy of systemic chemotherapy for advanced unresectable melanoma was assessed in terms of overall survival. Ten RCTs were included in the network of evidence (Figure 1) [18-27], which were identified with a systematic review of the literature. The treatments were categorized as dacarbazine monotherapy (DTIC), DTIC + Interferon (DTIC + IFN), DTIC + non-IFN, and Non-DTIC. Although the most recent treatments are not included, the analysis provides a useful example in oncology, where parametric survival analyses are often utilized.

### Network meta-analysis

The available survival data of the different studies was combined by means of a Bayesian NMA of parametric survival curves with models proposed by Jansen 2011 [15]. With this approach the survival of patients in a trial for the interventions being compared are modeled over time with parametric survival functions and the difference in the shape and scale parameters of these functions between interventions are synthesized and indirectly compared across trials. Within the Bayesian framework, analyses consist of data, likelihood, parameters, and a model. The data was extracted from the included RCTs, where for each arm the reported Kaplan Meier curves were digitized (DigitizeIt v1.6.1). A binomial likelihood distribution was used for the incident number of deaths for every two month interval, which was calculated based on the survival percentages from the Kaplan-Meier curves and the number of patients at risk at the beginning of the interval in each arm of each study, assuming a constant hazard rate within each interval (see Jansen and Cope [16] for more details). A two parameter Weibull NMA model was used with a random effect on the scale parameter [14-17] (See Additional file 1 for model details). Non-informative prior distributions were used for the model parameters to avoid influencing the results of the analysis based on prior beliefs. The parameters were estimated using a Markov Chain Monte Carlo within WinBUGS software [28], where inferences were based on 30,000 iterations from two chains and the first 30,000 iterations were discarded as ‘burn-in’.

## Results

### Treatment effects and functional estimates

*Relative treatment effects* of each intervention versus DTIC were expressed as HRs over time defined as:

(1) $$HR_{\mathrm{Ak}}\left(t\right)=\exp\left(d_{0\mathrm{Ak}}+{d_{1}}_{\mathrm{Ak}}\ln\left(t\right)\right)$$

where *HR*_*Ak*_(*t*) is the HR of intervention k relative to A (i.e. DTIC), and *d*_0*Ak*_ and *d*_1*Ak*_ are the differences in scale and shape of treatment k relative to A as obtained with the NMA.

**Figure 2:**
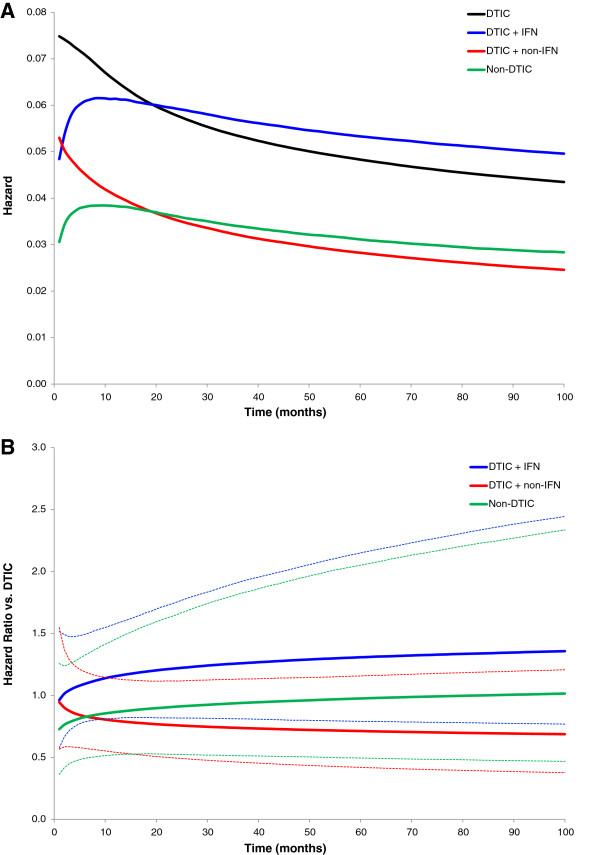
Hazard for each treatment over time (A), and Hazard ratio for each treatment versus DTIC over time (B).

**Figure 3:**
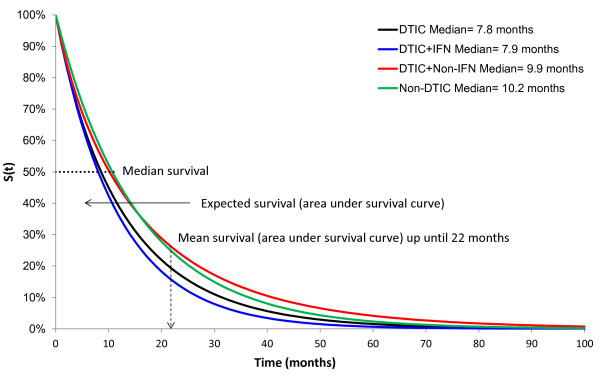
Survival proportions for each treatment over time.

In order to estimate the *hazard* and *cumulative hazard* function by treatment, the pooled differences in scale and shape were added to an average scale and shape for DTIC (obtained from the DTIC studies included in the NMA). These scale and shape estimates describe the hazard over time, as presented in Figure 2A. The ratio of hazard curves over time reflects the HRs, which are illustrated in Figure 2B, including the 95% credible intervals (dotted lines). The hazards (or HRs) were fairly constant over time, with some variation in the early months. The hazard over time for each treatment was transformed into *survival functions* as presented in Figure 3. Based on the survival functions, the *median survival* (i.e. time point where 50% of patients are still alive) as well as the *expected survival* (i.e. mean survival based on the area under the curve up to the time-point when all of the patients have died) were estimated. The area under the survival curve at the left of each time point represents the *mean survival* up until the corresponding follow-up time. This represents a summary measure of survival which does not require the curves to be fully extrapolated (i.e. up until when all patients have died). In the current evidence network, the *mean survival at 22 months* was assessed, which reflected the shortest follow-up across the studies, i.e. the DTIC arm in the study by Middleton et al. 2000. The median survival, mean survival at 22 months, and the expected survival are presented in Table 1.

**Table 1:** Overview of time-independent summary measures

**Outcome**	**DTIC**	**DTIC + IFN**	**DTIC + non-IFN**	**Non-DTIC**
Median survival	7.85	7.87	9.88	10.19
Expected survival (after all patients died) and 95% credible interval	12.61 (11.31, 14.13)	11.41 (8.44, 15.48)	16.11 (11.21, 23.14)	15.31 (9.17, 24.34)
Mean survival at 22 months and 95% credible interval	9.84 (9.13, 10.60)	9.61 (7.66, 11.72)	11.15 (8.88, 13.33)	11.23 (8.05, 13.99)

### Graphical and numerical summaries of rank probabilities

The ranking for all four treatments according to the probability of being the 1^st^, 2^nd^, 3^rd^, and 4^th^ best treatments was assessed on the basis of each of the aforementioned effect measures.

**Figure 4:**
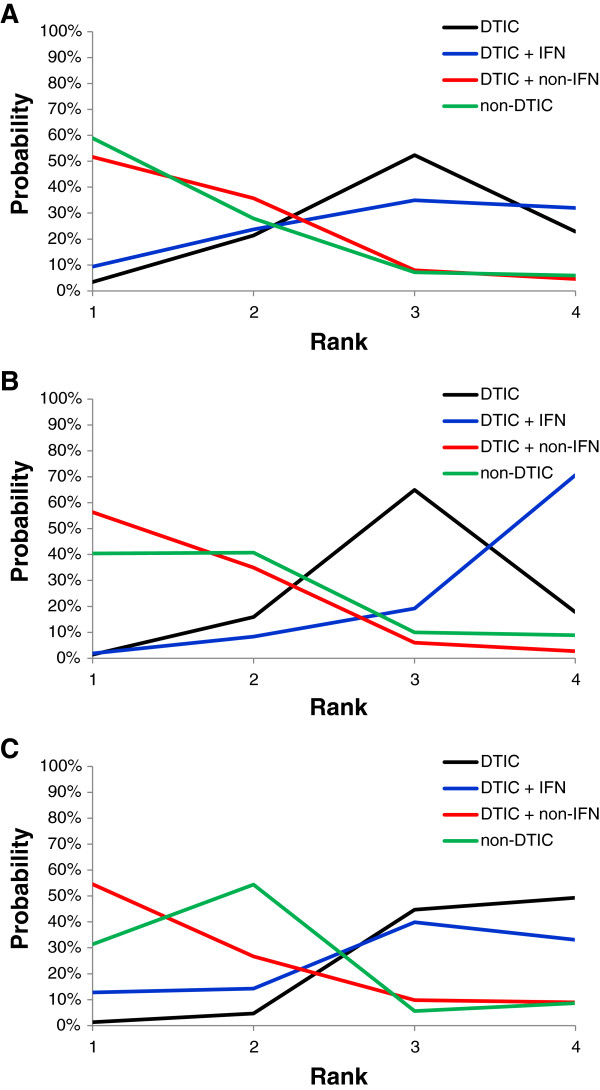
Rankograms: Probability plots for competing interventions based on median survival (A), expected survival (B), and mean survival at 22 months (C).

**Figure 5:**
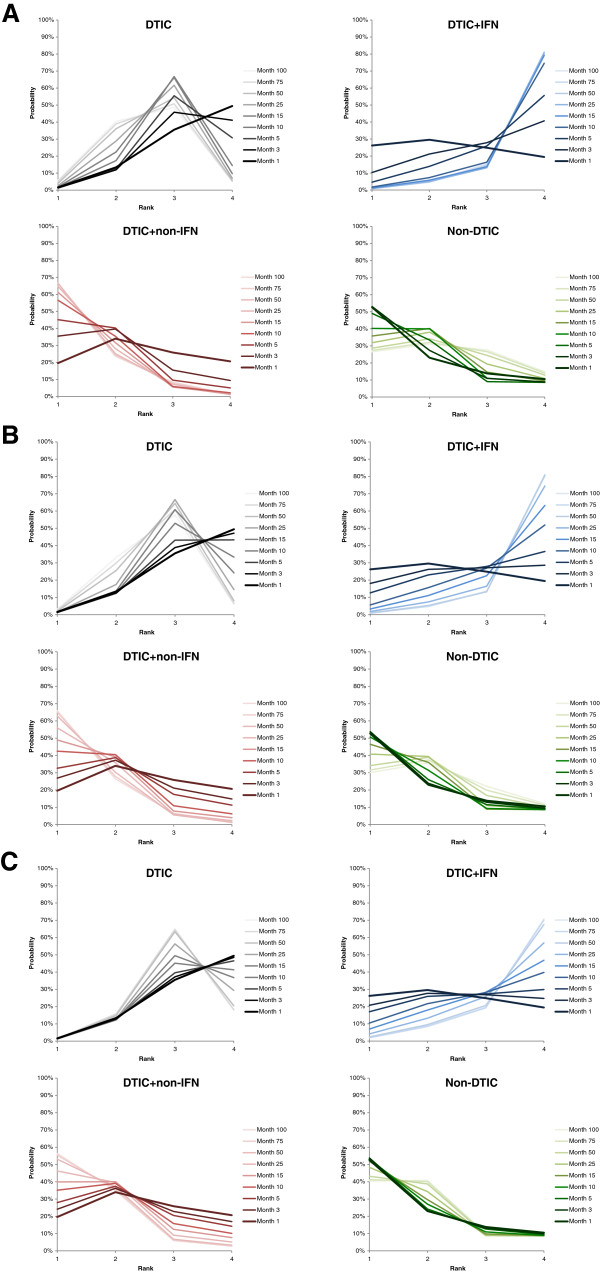
Probability plots for competing interventions based on hazard (ratio) over time (A), survival proportions over time (B), and mean survival over time (C).

*Rankogram*s, presenting the probability per rank for each treatment, are illustrated in Figure 4 for the time independent treatment effects, including median survival, expected survival, and mean survival at 22 months of follow-up. Rankograms for the time-varying treatment effects are presented in Figure 5, corresponding to the hazard (ratio), survival proportions, and mean survival.

The rankograms for the time-independent measures were fairly similar with some minor differences, suggesting DTIC + non-IFN and non-DTIC tended to have the highest probabilities of being the best and second best treatments. DTIC generally had the highest probability of being third best, and DTIC + IFN usually had the highest probability of being the worst. The rankograms for median survival (Figure 4A) and expected survival (Figure 4B) were mostly comparable (with a slight tradeoff between treatment ranks 3 and 4 for DTIC and DTIC + IFN), whereas the rankogram based on the mean survival at 22 months (Figure 4C) differed because the survival curves crossed at about 17 months for non-DTIC and DTIC + non-IFN.

The time-dependent measures were generally comparable and similar to the time-independent measures, indicating that DTIC + non-IFN and non-DTIC were the best treatments, followed by DTIC and DTIC + IFN. Variation in the hazard in the initial period is most obvious in Figure 5A, where the probability of being the best treatment is based on the HR. This indicates that non-DTIC is the best treatment for the first 5 months, after which time DTIC + non-IFN is the best treatment. The rankograms based on the survival proportions (Figure 5B) were similar to those based on the hazards, although the decrease in the probability of DTIC + IFN being the best (and second best) was less dramatic with the former. Rankograms based on the mean survival over time were also similar to those based on HRs and survival proportions, where results for DTIC differed the most which remained more consistent over time with respect to the probability of best and second best. Similarly rankograms for non-DTIC based on the mean survival were less sensitive to differences over time.

Another measure to summarize probabilities proposed by Salanti et al. [13] is the *surface under the cumulative ranking curve (SUCRA)*, which provides a summary statistic for the cumulative ranking. SUCRA ranges from 0 to 1, where 1 reflects the best treatment with no uncertainty and 0 reflects the worst treatment with no uncertainty. SUCRA for treatment *k* out of competing interventions *a* can be expressed as follows based on *a* vector of cumulative probability *cum*_*k*,*b*_ to be among *b* best treatments:

(2) $$\mathrm{SUCR}A_{k}=\frac{\sum_{b=1}^{a-1}\mathrm{cu}m_{k,b}}{a-1}$$

**Figure 6:**
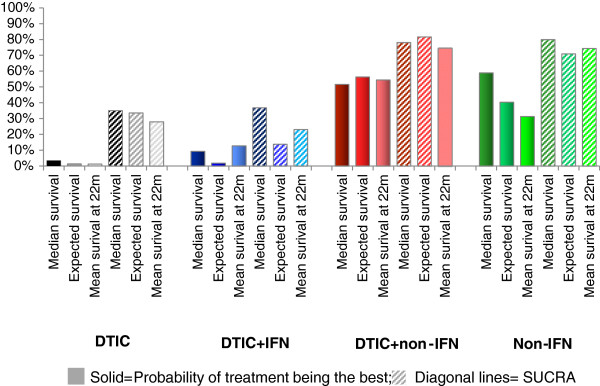
Probability of being the best treatment and SUCRA for median survival, expected survival, and mean survival at 22 months.

**Figure 7:**
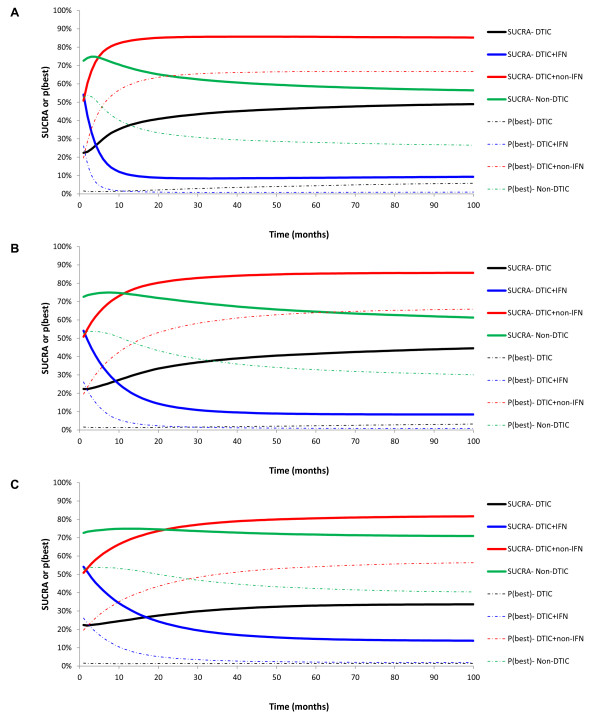
Probability of being best treatment and SUCRA for the hazard (ratio) over time (A), survival proportions over time (B), and mean survival at subsequent time points (C).

SUCRA was assessed for all effect measures for each treatment. In order to emphasize the importance of assessing SUCRA, probabilities of being the best treatment are compared to the SUCRA scores. Figures 6 and 7 present the probability that each treatment is best as well as SUCRA for the time-independent and time-dependent measures.

Figure 6 illustrates that the pattern associated with the probability of being the best treatment is fairly consistent with the results for SUCRA for the time independent measures. However, for non-DTIC, the probability of being best is lower than SUCRA for the mean at 22 months relative to the median and expected mean because SUCRA accounted for the higher probability of non-DTIC being the second best treatment. Generally, the difference between DTIC and DTIC + IFN was less pronounced for the probability of being the best treatment than for SUCRA, whereas the difference between DTIC + non-IFN and non-DTIC was more pronounced for the probability of being the best than for SUCRA.

The overall pattern for the probability of being the best treatment and SUCRA are similar for the time-independent and time-varying outcomes (Figure 7), although there were some differences depending on the specific time-varying measure. For example, differences between the probability of being the best treatment and SUCRA for DTIC + non-IFN and non-DTIC were greatest when based on the HRs and smallest when based on the mean survival. Additionally, the initial period where non-DTIC is expected to be the best treatment is shortest when based on the HR, longer when based on the survival function, and longest when based on the mean survival (up until almost 30 months). A unique feature of the rankograms based on survival is that the point at which the probability of being the best treatment switches from non-DTIC to DTIC + non-IFN is also the time point when the survival curves cross. As with the time-independent measures, for all three time-dependent measures the probability of being the best treatment suggests that DTIC and DTIC + IFN are comparable and reflect the two worst treatments, whereas DTIC + non-IFN appear to be better than non-DTIC. By evaluating SUCRA it is possible to differentiate DTIC and DTIC + IFN, where a majority of the time points suggest DTIC had a higher proportion than DTIC + IFN. Moreover, differences between DTIC + non-IFN and non-DTIC are less dramatic for SUCRA (as opposed to the probability of being the best treatment), particularly for mean survival. Overall, SUCRA results may raise questions about the additional efficacy of IFN in combination with DTIC as opposed to DITC alone.

## Discussion

### Advantages and disadvantages of different effect measures in relation to treatment ranking

Table 2 outlines the previously described alternative effect measures resulting from a NMA of survival data involving a multi-dimensional treatment effect. While these measures are all related and based on the same analysis, each measure involves a different interpretation and slightly different rank probabilities. Therefore, it is necessary to consider the advantages and disadvantages associated with each measure to calculate the rank probabilities.

**Table 2:** Summary of alternative methods for calculating rank probabilities

**Measure**	**Probability that a treatment is associated with:**	**Explicitly reflects time effect**	**Reflects cumulative effect over time**	**Requires baseline risk**	**Advantage**	**Disadvantage**
Median survival	The greatest survival time when 50% patients are alive	No	Yes	Yes	Commonly used and clinically relevant; Easily summarized as statistic; May limit need for extrapolation;	Ignores what happens after 50% of subjects have experienced the event;
Expected survival	The greatest expected survival	No	Yes	Yes	Directly relevant for cost-effectiveness; Easily summarized as statistic;	Sensitive to tail of distribution (may involve extrapolation); Does not illustrate time-varying results or time of greatest treatment effect; May not be as clinically relevant;
Mean survival at time t	Greatest mean survival (area under the curve) up until time t	No	Yes	Yes	Limits need for extrapolation if time t corresponds to follow-up time of trial with shortest duration; Easily summarized as statistic	May be difficult to interpret; Requires subjective selection of time t; Ignores tails of distribution and does not illustrate time-varying results;
Hazard (ratio) over time	The smallest hazard (ratio versus reference treatment) over time	Yes	No	Yes for hazard,	Directly relates to model and may help emphasize changes in treatment effect over time;	Does not capture cumulative effect of treatment over time; May lead to over interpretation near tail of distribution; Cannot be summarized as statistic (requires graphical illustration); May be more difficult to understand;
				No for hazard ratio		
Survival proportion over time (Cumulative hazard over time)	The greatest survival (proportion) over time	Yes	Yes	Yes	Highly intuitive and clinically relevant; Can be easily compared to data;	Cannot be summarized as statistic (requires graphical illustration);
Mean survival over time	Greatest mean survival (area under the curve) over time	Yes	Yes	Yes	Reflects a cumulative summary of survival proportions up until that time point, thereby de-emphasizing tail of distribution;	Cannot be summarized as statistic (requires graphical illustration); May be more difficult to understand;

### Time independent measures: median survival, expected survival, or mean survival at a specific time point?

Median survival provides an intuitive outcome for clinicians, which can easily be compared across treatments and is not very sensitive to parametric modeling assumptions. However, the median survival does not capture survival information beyond the time point at which 50% of patients have died, thereby providing a limited effect measure to rank treatments.

Expected survival is the primary measure of interest for cost-effectiveness evaluations involving survival, although this measure may require extrapolation of survival proportions beyond the follow-up of the included studies. Consequently, rank probabilities base on the expected survival may often rely on extrapolation, possibly to a different extent for each treatment. Also, althoug the estimates from a parametric model will reflect this uncertainty, the structural uncertainty regarding the choice of the underlying distribution is not captured, which is an important consideration [29].

Rank probabilities based on the mean survival at a specific time point, such as the duration of the trial with the shortest follow-up, may avoid extrapolation. Therefore this summary measure may be less sensitive to the assumptions of extrapolation, although a subjective choice regarding the time point for analysis is required, which leaves it open to criticism.

Overall, a summary measure such as the median survival, expected survival, or mean survival at a specific follow-up time has the advantage of providing a simple statistic that does not require graphical presentation over time. Consequently, rank probabilities based on these effect measures are therefore easier to interpret and compare across different analyses than time-varying effect measures. However, the rank probabilities associated with the one dimensional effect measures do not capture the possible time-varying nature of the underlying hazard of dying and are sensitive to the choice of effect measure.

### Time-varying measures: hazard, survival, or mean survival over time?

Rank probabilities based on the hazard or HR at each time point reflect the treatment ranking at each time point independent of previous time points. Presenting the hazard or HR over time illustrates how treatment effects may vary over time, which may not be easily detected based on the corresponding survival curves. However, HR curves may not be straightforward to interpret and later time points may be less relevant due to the small proportion of subjects that remain at risk of dying.

Rank probabilities based on the survival proportions reflect the cumulative treatment effect of the hazard up to that point in time. Survival curves can be considered the most complete and intuitive representation of treatment effects over time. Presenting survival proportions also allows the results of the meta-analysis to be compared to the observed survival curves reported for the individual studies. Therefore, rank probabilities presented over time based on survival curves may provide the simplest interpretation and the most ‘face validity’ as compared to those based on the hazards or HRs over time, especially considering that decision-makers are likely to be mostly concerned with actual survival over time, as opposed to the risk of dying at each time point.

Mean survival at subsequent time points may provide another measure with value as well for treatment rankings. By evaluating the area under the curve up to each time point, as opposed to the actual survival percentages, more weight is attributed to earlier treatment effects when a greater proportion of patients are still alive. If two treatments cross, the treatment with the more favorable survival in the beginning will result in a longer period of being the best treatment if the mean survival is used in comparison to the survival proportions or the HRs. Emphasizing the early treatment effects (and de-emphasizing later ones) may be considered useful given the increasing uncertainty in treatment effects over time due to reduced population at risk and the possible extrapolation of survival curves.

Overall, rank probabilities of treatment effects over time may provide a more transparent and informative approach to help guide decision-making in comparison to single rank probabilities based on collapsed measures, such as median survival, or expected survival. Rank probabilities based on survival proportions may be the most intuitive and straightforward to communicate, but alternatives based on the hazard function or mean survival over time may be useful as well.

### Probability of being best treatment, rankograms, or SUCRA?

Summarizing treatment effects and their associated uncertainty in terms of the probability that each treatment is best is often presented, although when there is considerable variation in the uncertainty regarding the relative treatment effects, this approach may lead to false conclusions. Rankograms provide the most informative and balanced approach to translate treatment effects and their associated uncertainty into probability statements for decision-making by presenting the probability that each treatment is best, 2^nd^ best, 3^rd^ best, etc. However, rankograms become more difficult to interpret for time-varying treatment effects as compared to one-dimensional effect measures. In the context of time-varying treatment effects, graphing SUCRA may provide a more concise summary measure than presenting all rank probabilities.

## Conclusion

In this paper we present different alternatives for quantitative summaries of treatment effect estimates obtained with NMA of survival data to help inform decision-making. Rank probabilities based on one-dimensional measures such as median survival, expected survival, or mean survival at one follow-up time are relatively easy to understand, but do not provide the wealth of information captured by rank probabilities over time. Survival proportions reflect the cumulative effect of treatments over time and provide the most intuitive basis for rank probabilities and SUCRA. Rank probabilities based on the hazard (ratio) function over time provide information on the treatment effect at each time point, ignoring effects at previous time points. Rank probabilities based on the mean survival at each time point give more weight to the treatment effects when a greater proportion of patients are alive. Rankograms of time-varying treatment effects can be presented efficiently with SUCRA.

## Competing interests

The authors declare that they have no competing interests.

## Authors’ contributions

SC and JJ conceived of the study. SC performed the statistical analysis and drafted the manuscript. JJ participated in the study design and helped to draft the manuscript. All authors read and approved the final manuscript.

## Pre-publication history

The pre-publication history for this paper can be accessed here:

http://www.biomedcentral.com/1471-2288/13/147/prepub

## Supplementary Material

Additional file 1 **Appendix.** Random effects first order fractional polynomial network meta-analysis model for survival curves. Click here for file
