# Supplementary material for: Quantitative summaries of treatment effect estimates obtained with network meta-analysis of survival curves to inform decision-making
Source: BMC Med Res Methodol. 2013 Dec 1;13:147. doi: 10.1186/1471-2288-13-147 (PMC3866977; doi:10.1186/1471-2288-13-147)
Supplement: Additional file 1 — Appendix. Random effects first order fractional polynomial network meta-analysis model for survival curves. [file 1471-2288-13-147-S1.docx]

**Appendix:** **Random effects first order fractional polynomial network meta-analysis model for survival curves**

When outcome data is available at multiple time points, fractional polynomial based (network) meta-analysis models can be used [Jansen, 2011]:

(1)

$\theta_{jk}$reflects the ‘underlying’ outcome for treatment *k* in study *j* at time point *t* and the link function to transform this outcome to a normally distributed scale. The outcome is now described as a function of time *t* with $p=\{-2,-1,-0.5,0,0.5,1,2,3\}\text{ and }t^{0}=\text{ln(t)}$ and with treatment and study specific ‘scale’ and ‘shape’ parameters $\beta_{0jk}$and $\beta_{1jk}$. For the current study p=0 was used, which translated to ln(t) or the ‘Weibull’ model. The vectors $\left(\begin{matrix} \mu_{0jb} \\ \mu_{1jb} \end{matrix}\right)$are trial-specific and reflect the true underlying scale and shape parameters of the comparator treatment *b*. $\delta_{0jbk}$is the study specific difference in the scale parameter$\beta_{0}$ , i.e. the difference in the intercept of the ‘outcome curves’ for treatment *k* relative to comparator treatment *b.* $\delta_{0jbk}$are drawn from a normal distribution with the pooled estimates expressed in terms of the overall reference treatment *A*: $d_{0Ak}-d_{0Ab}$with $d_{0AA}=0$. The pooled difference in the shape parameter$\beta_{1}$for treatment *k* relative to comparator treatment *b* is also expressed as $d_{1Ak}-d_{1Ab}$. By incorporating$d_{1Ak}$ in addition to$d_{0Ak}$, a multi-dimensional treatment effect is used.

Variance$\sigma^{2}$reflects the heterogeneity in the difference in the scale parameters across studies. A random effects model with only a heterogeneity parameter for $d_{0Ak}$ implies that the between study variance of the log hazard ratios remains constant over time.
